# Sustained circulation of yellow fever virus in Cameroon: an analysis of laboratory surveillance data, 2010–2020

**DOI:** 10.1186/s12879-022-07407-1

**Published:** 2022-04-29

**Authors:** Fredy Brice Simo Nemg, Ngu Njei Abanda, Martial Gide Yonga, Diane Ouapi, Ivis Ewang Samme, Marlise Dontsop Djoumetio, Marie Claire Endegue-Zanga, Maurice Demanou, Richard Njouom

**Affiliations:** 1 Department of Biochemistry, University of Yaounde 1, Yaounde, Cameroon; 2 Virology Unit, Virology Service, Centre Pasteur of Cameroon, 451 Rue 2005, BP 1274 Yaounde, Cameroon; 3 Expanded Programme on Immunization, Ministry of Public Health, Yaoundé, Cameroon; 4 World Health Organization, Country Office, Yaounde, Cameroon; 5 World Health Organization, Inter-Country Support Team West Africa, 03 BP 7019 Ouagadougou, Burkina Faso

**Keywords:** Yellow fever, Cameroon, Surveillance, Yellow fever serology, Yellow fever high risk districts

## Abstract

**Background:**

The re-emergence of yellow fever poses a serious public health risk to unimmunized communities in the tropical regions of Africa and South America and unvaccinated travelers visiting these regions. This risk is further accentuated by the likely spread of the virus to areas with potential for yellow fever transmission such as in Asia, Europe, and North America. To mitigate this risk, surveillance of yellow fever is pivotal. We performed an analysis of laboratory-based surveillance of yellow fever suspected cases in Cameroon during 2010–2020 to characterize the epidemiology of yellow fever cases and define health districts at high risk.

**Method:**

We reviewed IgM capture ELISA and plaque reduction neutralization test (PRNT) test results of all suspected yellow fever patients analyzed at Centre Pasteur of Cameroon, the national yellow fever testing laboratory, during 2010–2020.

**Results:**

Of the 20,261 yellow fever suspected patient’s samples that were tested, yellow fever IgM antibodies were detected in 360 patients representing an annual average of 33 cases/year. A major increase in YF IgM positive cases was observed in 2015 and in 2016 followed by a decrease in cases to below pre-2015 levels. The majority of the 2015 cases occurred during the latter part of the year while those in 2016, occurred between February and May. This trend may be due to an increase in transmission that began in late 2015 and continued to early 2016 or due to two separate transmission events. In 2016, where the highest number of cases were detected, 60 health districts in the 10 regions of Cameroon were affected with the Littoral, Northwest and, Far North regions being the most affected. After 2016, the number of detected yellow fever IgM positive cases dropped.

**Conclusion:**

Our study shows that yellow fever transmission continues to persist and seems to be occurring all over Cameroon with all 10 regions under surveillance reporting a case. Preventive measures such as mass vaccination campaigns and routine childhood immunizations are urgently needed to increase population immunity. The diagnostic limitations in our analysis highlight the need to strengthen laboratory capacity and improve case investigations.

**Supplementary Information:**

The online version contains supplementary material available at 10.1186/s12879-022-07407-1.

## Introduction

Yellow fever (YF) continues to pose a serious threat to communities in the tropical regions of Africa and South America and unvaccinated travelers visiting these regions [1]. According to a recent modeling study, in 2018, there were 109,000 (95% credible interval (CrI) 67,000–173,000) YF infections resulting in 51,000 (95% CrI 31,000–82,000) deaths and nearly 90% of cases and deaths occurred in Africa [2]. YF is an acute viral hemorrhagic disease caused by an infection with the yellow fever virus (YFV). The virus is transmitted by certain species of *Aedes* and *Haemagogus* mosquitoes [3]. Clinically, most infected patients will be asymptomatic or present with mild febrile illness, body pain and weakness. However, about 20% of infected patients will progress to a more severe form of the disease characterized by high fever, bleeding, jaundice, shock and, multiorgan failure and about 50% of these severe patients will die [4].

A safe and effective YF vaccine exists and is distributed in endemic countries through routine childhood immunization and during preventive mass vaccination campaigns. Nonetheless, YF continues to cause disease including sporadic outbreaks. Between 2010 and 2020, 78 outbreaks in 28 countries were reported to WHO [5] and some of these outbreaks have been deadly. Such as the 2016 outbreak in Angola and the Democratic Republic of Congo that resulted in 7334 suspected cases and 393 deaths [6, 7]. The continuous transmission of YF could result from several factors but the most likely are the presence of a wildlife reservoir [8] and an increase in the density and distribution of the YF mosquito vector [9, 10]*.* YFV has a well-recognized wildlife (sylvatic) cycle involving non-human primates and forest-dwelling mosquitoes. This wildlife cycle maintains the virus in the forest habitat and humans that encroach these habitats may become infected. When these infected humans return to urban environments, the spread of YF is sustain in the human population by the urban mosquito vector, *Aedes aegypti* [11]. *Ae. aegypti* is well-adapted to the urban environment; it preferentially feeds on human blood, bites during daylight hours, breeds in small volumes of water found in plastic containers, tins and tires and survives dry severe conditions [12]. *Ae. aegypti* is widely present in urban areas including non-endemic YF areas. With the changing epidemiology of *Ae. aegypti*, there is concern that YF could erupt and spread beyond endemic areas [13].

Cameroon, a central African country, has experienced several YF epidemics [14, 15]. The first large, reported epidemic occurred in 1990 in the Far North region of the country. Although 182 cases and 125 deaths were reported, the actual numbers are estimated at 20,000 cases and 1000 deaths [16, 17]. In 2002, Cameroon enacted a strategy for the control YF with prevention activities such as routine childhood immunization and mass vaccination campaigns in high-risk health districts identified through case-based surveillance of YF. Once a suspected case of YF is confirmed within a health district, a case-response investigation is initiated within the health district. The case-response investigation assesses and responds to the outbreak with both emergency measures and preventive immunization plans [18]. Here, we analyze laboratory-based surveillance data of YF suspected patients collected during 2010–2020 to characterize the epidemiology of YF cases and the proportion of high-risk health districts in Cameroon.

## Methods

### The National Yellow Fever (YF) surveillance system in Cameroon

YF surveillance in Cameroon is planned and implemented by the Expanded Programme on Immunization (EPI) of the Ministry of Public Health with support from developmental partners [18]. Generally, health facilities are required to identify and report patients that match the standard WHO definition of a suspected YF case. That is a patient with an illness characterized by an acute onset of fever followed by jaundice within 2 weeks of the onset of the first symptom [19]. Once a patient is identified, the health facility is required to fill a case investigation form with the patient’s demographic and clinical information and collect a venous blood sample. The collected blood sample is centrifuged, and the resulting plasma/serum is collected. The case report and the collected plasma/serum sample are sent to the EPI through the health district. The EPI then transports the plasma/serum sample to Centre Pasteur du Cameroun (CPC) for laboratory testing. In Cameroon, all laboratory testing of YF suspected samples is performed at CPC. CPC is accredited by the WHO Global YF Laboratory Network and has served as the Regional YF reference laboratory since 2019. As soon as the laboratory confirms the case, the EPI and the health district with the laboratory confirmed case then cooperate and initiate a case-response investigation within that health district [18, 19]. CPC has routinely registered laboratory data from YF surveillance since 2000.

### Laboratory testing

Laboratory testing is required to confirm a case of YF. Laboratory confirmation is based on one of the following criteria: the presence of YF virus RNA in blood from a person with no history of recent YF vaccination or presence of YF virus-specific IgM antibody and absence of other relevant flaviviruses (Dengue virus, West Nile virus, Zika virus) in person with no history of yellow fever vaccination [20].

Plasma/serum samples collected ≤ 10 days from symptom onset were tested for YF viral genome by RT-PCR as previously described [21]. All samples were tested for YF virus-specific IgM using a modified IgM antibody Capture ELISA assay protocol of the U.S. Centers for Disease Control and Prevention (CDC) [19]. Briefly, 96-well microtiter plates were coated with affinity purified goat anti-human IgM antibodies (Kirkegaard and Perry laboratories Inc.) in bicarbonate buffer and incubated overnight 4 °C. The coated plates were blocked with 10% (v/v) fetal bovine serum in PBS/Tween/skim milk for 1 h at 37 °C. The plates were then washed, and heat inactivated serum/plasma samples diluted 1:100 in PBS containing 0.05% Tween plus 1% skim milk powder (ELISA Immunoassay buffer (EIA buffer)) was added. After 1 h incubation at 37 °C, unbound antibodies were washed away and YFV antigen (obtained from Institut Pasteur Dakar, Senegal) diluted in EIA buffer was added. After 1 h of incubation at 37 °C in a moist chamber, the plates were washed to remove excess antigen. A commercially available flavivirus group reactive monoclonal antibody 6B6C-1 horseradish peroxidase conjugated (Hennessy Research, USA) was added to each well of the plates. Plates were then washed to remove excess conjugate before they were developed with TMB for 5 min at room temperature. The reaction was stopped by adding 100 μl of 1 N H_2_SO_4_. The ODs were read at 450 nm (with 620 nm reference filter) with an automated microplate reader (Biorad PR 3100 TSC Microplate, USA). The positive-to-negative (P/N) ratios were determined, and a sample was considered positive for YF virus IgM if its P/N ratio was ≥ 2.0 and the difference between the mean OD of test sample reacted with YF viral antigen and the mean OD of the test sample reacted with normal antigen was > 0.2 (Pt > 0.2). All positive samples were retested and if found to be negative, were reported as indeterminate. Also, positive, and indeterminate samples were first screened for other flaviviruses (Dengue virus, West Nile virus and Zika virus) using an in-house flavivirus differential IgM antibody Capture ELISA assay and then confirmed with 50% plaque-reduction neutralization tests (PRNT). PRNT assay was adapted from previously described [22, 23] using the YF17D virus strain. Briefly, samples were heat-inactivated at 56 °C for 30 min and diluted 1:10 in Leibovitz L15 culture medium. Serial two-fold dilutions of samples were then prepared from the 1:10 dilution. An equal volume of YFV-17D vaccine, calculated to yield approximately 10^3^ PFU/ml, was added to each sample dilution, and incubated in a 24 well plate at 37 °C for 1 h. Confluent stable porcine kidney epithelial (PS) cells (10^6^ cells/ml) were then added to each well of the virus-sample mixture and mixed. The 24-well plate containing virus-sample and confluent cells was incubated at 37 °C for 4 h in an incubator to allow for virus absorption. The inoculated plates were then overlaid with carboxymethylcellulose (CMC) diluted in L15 medium and placed at 37 °C in an incubator for 5 days. After 5 days of incubation, the CMC medium was discarded, the cell monolayers were stained with amido black, and plaques were counted. The neutralizing antibody titer was identified as the highest serum dilution that reduced the number of virus plaques in the test by 50%.

Samples received between April and December of 2018 were tested using a new YF IgM antibody capture ELISA assay kit (YF MAC-HD) developed by the CDC [24]. Due to reagent shortages, testing with this new kit was halted and reverted to the modified IgM antibody Capture ELISA test described above. Prior to 2020, all YF IgM positive samples identified in the laboratory were shipped to the WHO Regional Reference Laboratory at Institut Pasteur de Dakar, Senegal, for confirmatory testing. Also, for quality control purposes, 10% of YF IgM negative samples received in the laboratory each semester were shipped to the WHO Regional Reference Laboratory for testing. The agreement in test results of the 10% of YF IgM negative sera shipped to the Regional Reference Laboratory was always greater than 90%.

### Collection of data, mapping, and statistical analysis

Demographic data for all YF suspected samples received in the laboratory during 2010–2020 were extracted retrospectively from the YF database. The nonparametric Mann–Whitney test was used for comparisons of means of continuous variables and the chi-square test was used for comparison of categorical variables. Microsoft Excel and Graphpad Prism version 9.1.2 for Windows (GraphPad Software, California, USA) were used for statistical analysis. The map of YF IgM positive cases was constructed using the QGIS Geographic information system software (QGIS version 3.18.1-Zurich, QGIS Development Team). The shape files of the Map of Cameroon including health regions and districts used in the QGIS software was developed by the National Institute of Cartography of Cameroon.

## Results

### Characteristic of YF suspected patients

During the period 2010 to 2020, 21,800 plasma or serum samples were received from patients that met the WHO YF case definition. Due to periodic reagents stockouts during the study period, 1539 samples received were not tested and excluded from further analysis. Accordingly, a total of 20,261 YF suspected patient samples were tested during the study period (Table 1). Overall, YF suspected patients had an average age of 19.2 years (standard deviation of 16.9). A higher proportion of the YF suspected patients were males 61% (12,301/20,261) and unvaccinated 75% (15,094/20,261). Baseline characteristics were similar among YF positive and YF negative patients with the exception that YF positive patients were more likely adults (p < 0.0001) and unvaccinated (p = 0.0011) (Table 1).

**Table 1:** Demographic characteristic of suspected Yellow Fever (YF) patients received in the laboratory from 2010 to 2020

Characteristic	YF positive (N = 360)	YF negative (N = 19,901)	*P value	All patients (N = 20,261)
Age (y); mean ± SD	22.6 ± 16.4	19.1 ± 16.9	P < 0.0001	19.2 ± 16.9
Age groups				
Pediatric (0–14y)	127	9774	P < 0.0001	9901
Adults (15–64y)	226	9709		9935
Geriatric (≥ 65y)	7	418		425
Gender				
Male	226	12,075	P = 0.445	12,301
Female	133	7777		7910
Unknown	1	49		50
Vaccination status				
Vaccinated	119	5048	P = 0.0011	5167
Unvaccinated/	241	14,853		15,094

*Results having a P value < 0.005 were considered statistically significant

### Annual number of YF positive cases

**Fig. 1:**
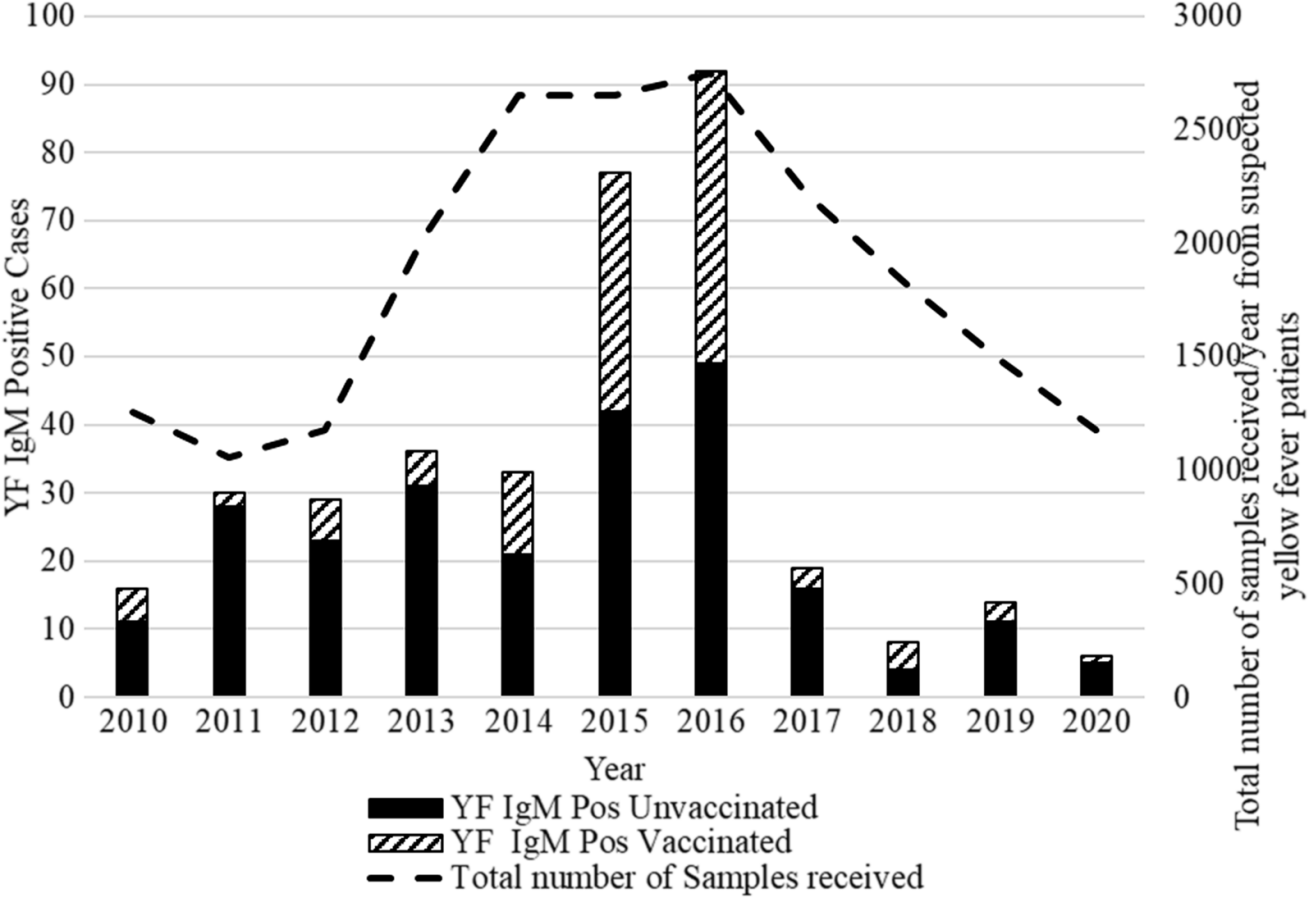
Annual number of Yellow Fever (YF) IgM positive cases, Cameroon, 2010–2020

RT-PCR testing of samples ≤ 10 days from symptom onset began in 2020. As such, only 2020 samples that met the criteria of samples collected ≤ 10 days from symptom onset were tested by RT-PCR for YF RNA. All samples tested by RT-PCR yielded negative results. However, all samples were tested with the YF IgM capture ELISA test. The IgM ELISA test revealed 339 positives, 19,887 negatives and 35 indeterminate. Of the 35-patient sample with indeterminate results, 21 had a positive confirmatory PRTN 50 result. Not all YF IgM ELISA positive cases were confirmed with the PRNT assay (i.e., not tested with the PRNT assay) as recommended by the testing algorithm [20] due to unavailability of testing reagents (Additional file 1: Table S1). As such, all 339 IgM positive and the 21 indeterminate cases with confirmatory PRNT 50 positive samples were considered as YF IgM positive cases and included in the analysis. On average, 33 YF IgM positive cases were detected annually during the study period and the number of cases varied from 6 to 92. Among the YF IgM positive unvaccinated cases (N = 241), the number of cases increase from 2011 and peaked in 2016 and decreased then after (Fig. 1).

### Seasonality

**Fig. 2:**
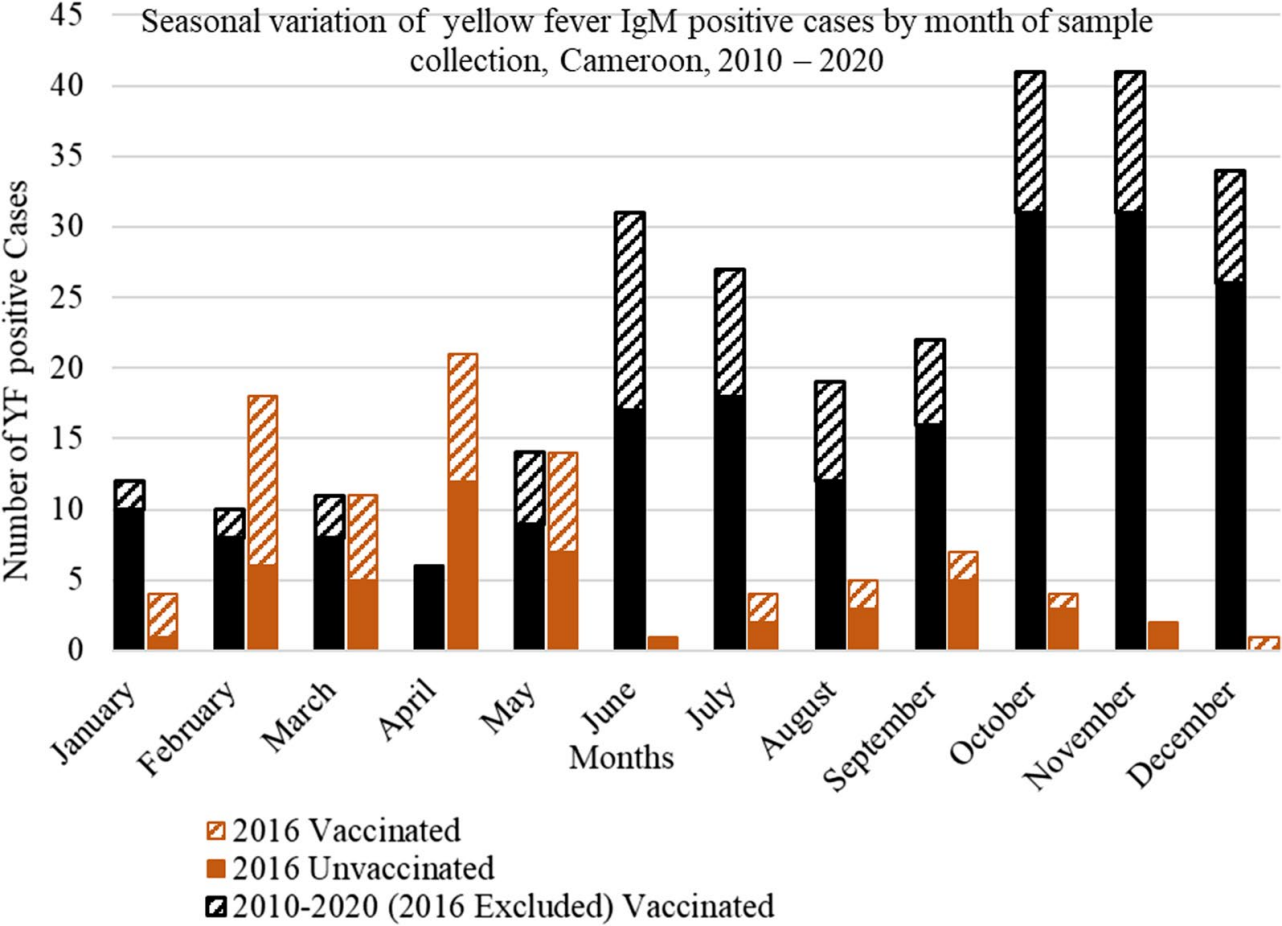
Seasonal variation of yellow fever IgM positive cases by month of sample collection, Cameroon, 2010–2020

The distribution of YF positive cases by month is reported in Fig. 2. Although the diagnosis YF IgM positive cases had no well-defined peak month or periods, we observed that in general, YF IgM positive cases increased in October and November and declined sharply by January. However, we observed a different trend in the distribution of YF IgM positive cases in 2016. In that year, the cases increased between February and May and declined for the rest of the year.

### Geographic distribution of YF IgM positive cases

**Fig. 3:**
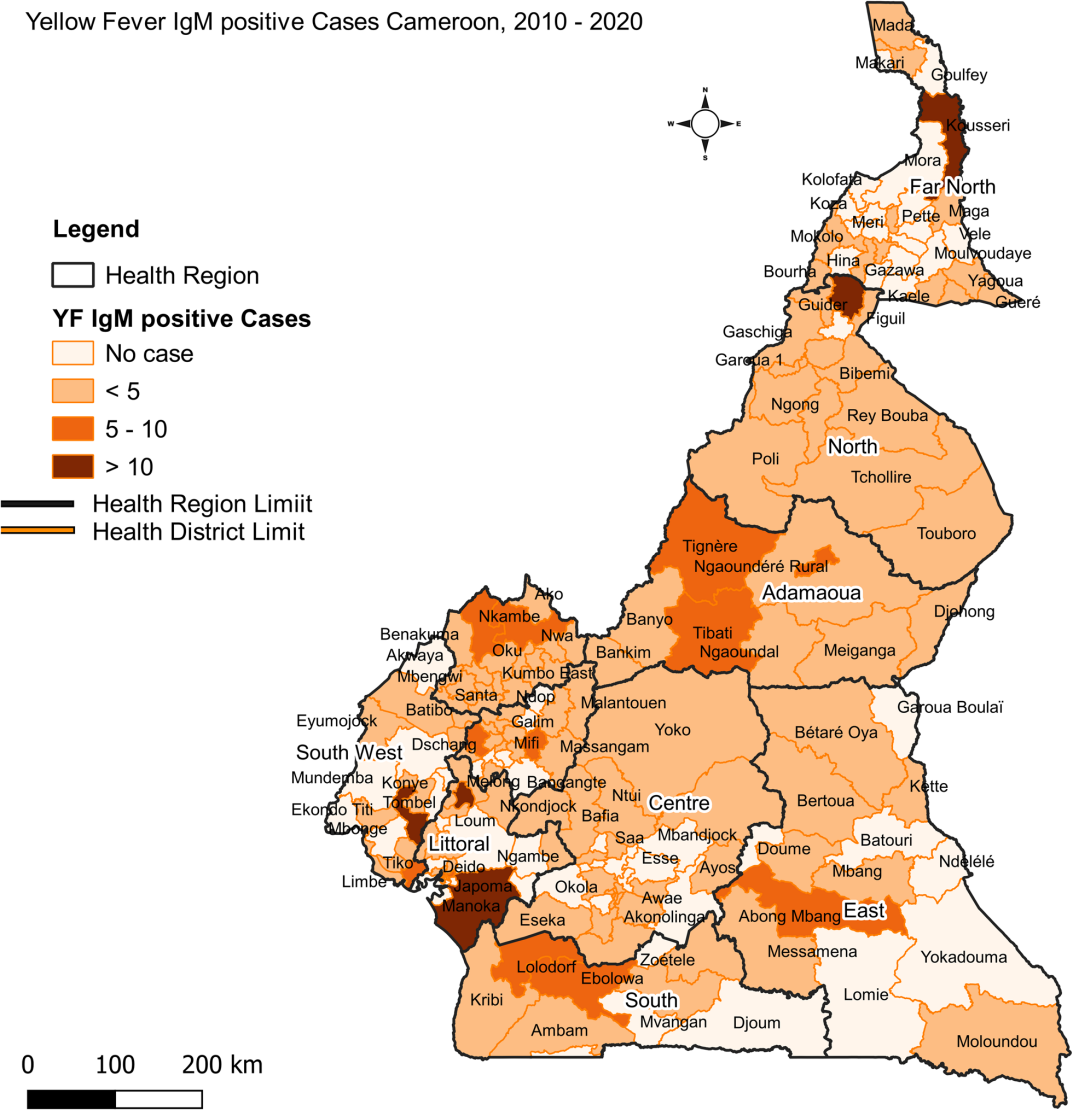
Map of spatial distribution of yellow fever IgM positive cases, 2010–2020 Cameroon

Throughout the study period, YF IgM positive cases were reported in all 10 regions of Cameroon. A total of 131 out of 197 health districts in Cameroon reported at least one YF IgM positive case. The proportion of health districts with at least one positive case per year increased from 15 in 2010 to 60 in 2016 and then declined then after to 14 in the subsequent year and 6 in 2020. Figure 3 shows the distribution of YF IgM positive cases per health district and region. Amongst the regions, the Littoral region reported the highest number of positive cases (61) followed by the North (47) and Northwest (45) regions, respectively. Guider health district in the North region reported the highest number of positive cases (17) but the majority of the cases occurred in 2011. Health districts that reported higher numbers of positive cases (> 5) in the last 5 years include the health districts of Kousseri and Nkongsamba. Both health districts reported 6 cases in 2016. After 2016, health districts were reporting only 1 to 3 cases often in the same year or spaced out over the years (Additional file 2: Table S2).

## Discussion

In this analysis of laboratory-based surveillance data, YF IgM antibodies were detected in 360 YF suspected patients during the 2010–2020 period, representing an annual average of 33 cases/year. The annual number of reported YF suspected cases increased from 2013, peaked in 2016 and declined then after. A major increase in the number of YF IgM positive cases was observed in 2015 and 2016 followed by a decline to below pre-2015 levels. This increase in YF IgM positive cases most likely reflects an increase in transmission rather than an increase in the number of cases tested as a similar number of samples were tested in the preceding year. A similar pattern was also observed with the number of affected health districts. The number health districts with a YF IgM positive case almost doubled in 2015 and peaked in 2016 affecting 60 health districts in the 10 regions of Cameroon. In 2016, the three regions Littoral, Northwest and Far North regions accounted for 50% of the number of cases. Furthermore, the monthly distribution of YF IgM positive cases in 2016 differed from those of 2015 and other years with a peak in the number of cases occurring between February and May and remaining low for the rest of the year. This surge in cases in early 2016 suggest an increase in transmission that could have begun in late 2015 and continued to early 2016. However, the low number of cases observed in January, suggest two separate transmission events might have occurred (Additional file 3: Table S3). This major increase and monthly distribution of YF IgM positive cases between 2015–2016, emulates the 2015–2016 YF outbreaks in the Democratic Republic of Congo and Angola [25, 26]. Despite the surge in the number of YF IgM positive cases in 2015 and in 2016, no preventive mass vaccination campaign was organized [27]. Between 2010 and 2016, two YF preventive mass vaccination campaigns were organized in Cameroon. The first one was in September 2013 and involved 13 health districts in the Littoral region. During that campaign, over 850,000 people aged > 9 months were vaccinated [28]. The second, was a complementary preventive mass vaccination campaign conducted from the 26th to the 31st of August 2014. This campaign involved 27 health districts in the West and Southwest regions and targeted over 1.8 million persons [29]. Surprisingly, the Littoral region that benefitted from that mass vaccination campaign in 2013 was the hardest hit region in 2016 raising concerns about the effectiveness of the campaign. After 2016, there was a sharp reduction in the number of YF positive cases as well as a reduction in the number of reported suspected cases. Considering that there was no YF preventive vaccination campaign in 2015 and 2016 [27], it is unknown if this decline in cases resulted from reduced disease transmission or reduced disease surveillance activities.

According to a recent population-wide YF vaccination coverage study, Cameroon is assumed to have a moderately high (60–70%) YF vaccine coverage across all age groups [30]. This YF vaccination coverage study also estimates that only about 3 million individuals are required to be vaccinated for Cameroon to achieve the 80% population coverage threshold recommended by WHO to prevent outbreaks in all districts in Cameroon [30]. The accuracy of this study is uncertain, and the YF vaccination coverage levels could be misleading. First, routine infant YF vaccination began in Cameroon in 2004 [18] and this strategy needs time to affect population level coverage. Secondly, mass preventive campaigns targeting all age groups are infrequently organized and target small fractions of the population [18, 28, 29]. Lastly, assuming that the YF vaccine affords protection to all vaccinees [31], with such a high vaccine coverage, we will expect fewer outbreaks (Outbreak threshold = 1 laboratory confirmed case). However, and as portrayed by our data, the transmission of the YF virus continues to persist and seems to be occurring all over the country. Although the presence of the YF vector and reservoir are likely to sustain transmission, an optimal YF vaccine coverage is absolute necessary. An optimal vaccine coverage will prevent progression to severe disease and interrupt transmission. During the study period, 131 of the 197 health districts under surveillance reported at least one YF IgM positive case and about 40% of health districts reported a YF case in more than one year. This continuous transmission of the YF virus underlines the need for the EPI to strengthen vaccine coverage and surveillance activities.

The transmission of YF may be influenced by the climatic changes, environmental changes and population determinants [32]. Despite the varying climate and environmental conditions in Cameroon [33, 34], a YF case was reported in every region of the country. A possible reason for this could be the adaptation of the YF vector, *Aedes species* to the different climates and ecology in Cameroon [35]. Also, irrespective of the region, the risk of a YF infection seems to be elevated in October and November. This period corresponds to a transition from the wet to the dry seasons and this change in climate may affect the abundance and distribution of the yellow fever vector thereby increasing the possibility of infections. However, the year 2016 presented a unique trend with an increase in cases occurring early in the year between February and May and staying low for the rest of the year. This early 2016 surge could have begun in late 2015 but the lower number of cases detected in January of 2016 suggest a separate event. The reasons for the observed trend in 2016 are largely unknown. [34].

There are at least four limitations to findings in this study both related to surveillance activities and diagnosis. Primarily, our analysis relies solely on laboratory-based data and does not include case-response data from field investigations. Whenever a case is confirmed by the laboratory, a case-response investigation should be initiated by EPI and the health district to identify other cases or the extent of the transmission [26]. Unfortunately, case-response investigations are not regularly done due to budgetary constraints. Furthermore, even when investigations are performed, samples are generally not collected. As such, the extend of transmission and the true burden of YF infections are unknown. Secondly, in our analysis, we considered all YF IgM positive individuals both vaccinated and unvaccinated as YF positive cases. Of the 360 cases, 119 cases reported to have received a YF vaccine with vaccination dates to sample collection dates ranging from days to years. The IgM ELISA test cannot differentiate between YF IgM induced by vaccination and that induced by natural infection with the YF virus [20] and vaccinees could have YF IgM antibodies 3–4 years following vaccination [36]. As such, the YF laboratory results of the vaccinated individuals must be interpreted with care. Unfortunately, majority of the YF positive declared vaccinated cases were not investigated to confirm vaccination status. Nonetheless, in our figures we have distinguished vaccinated cases from unvaccinated cases. Thirdly, of the over 1000 samples received and tested each year, only about 2% have detectable YF IgM antibodies. Using the IgM ELISA test as a primary assay for laboratory detection, there is a possibility that several YF cases are likely missed. Niedrig and colleagues found that individuals with previous flavivirus exposure who received the YF vaccine failed to produce detectable IgM antibodies [37]. Cameroon is endemic to several flaviviruses [38–41] and the persistent exposure to flaviviruses could affect IgM stimulation in YF infected persons. However, it is unknown if the observation of Niedrig and colleagues in YF vaccinees also occurs during a natural infection with YF virus [37]. Lastly, our analysis is largely based IgM positive cases. IgM results are preliminary and are required to be confirmed by the PRNT assay. As shown in Additional file 1: Table S1, PRNT results were not available for all IgM positive cases. Despite these limitations, analysis of laboratory-based data confirms the continuous circulation of YF virus in Cameroon. To successfully prevent and control YF in Cameroon, all partners need to cooperate to reinforce control and prevention measures. Preventive measures such as mass vaccination campaigns and routine childhood immunization are urgently needed to increase population immunity. Furthermore, we greatly welcome the creation of the “Eliminate Yellow Fever Epidemics” (EYE) initiative. The EYE initiative is a bold and innovative strategy with three primary objectives: protect at-risk populations; prevent international spread of YF virus; and rapidly contain YF outbreaks. A core activity of EYE initiative is to strengthen laboratory diagnostic capacity in most YF endemic countries and to vaccinate over one billion people in YF endemic countries by 2026 [42].

## Conclusion

Our analysis of the laboratory surveillance data shows that yellow fever transmission continues to persist and seems to be occurring all over Cameroon with all 10 regions under surveillance reporting a case. Preventive measures such as mass vaccination campaigns and routine childhood immunizations are urgently needed to increase population immunity. The limitations in our analysis highlight the need to greatly strengthen laboratory diagnostic capacities in YF endemic countries and improve case investigations.

## Supplementary Information

**Additional file 1: Table S1.** Yellow fever IgM positive cases with PRNT results.

**Additional file 2: Table S2.** Yellow fever IgM positive cases per health district.

**Additional file 3: Table S3.** Yellow fever IgM positive cases per year and per month.

## Data Availability

The data that support the findings of this study are available from Centre Pasteur of Cameroon, and the Ministry of Public Health in Cameroon but restrictions apply to the availability of these data, which were used under license for the current study, and so are not publicly available. Data are however available from the corresponding author on reasonable request and with permission of Centre Pasteur of Cameroon. We developed the map depicted in Fig. 3.

## Abbreviations

YF: Yellow Fever
YFV: Yellow Fever Virus
CPC: Centre Paster of Cameroon
IgM: Immunoglobulin M
PRNT: Plaque reduction neutralization assay
ELISA: Enzyme-linked immunosorbent assay
